# Genotype Directed Therapy in Murine Mismatch Repair Deficient Tumors

**DOI:** 10.1371/journal.pone.0068817

**Published:** 2013-07-23

**Authors:** Melanie H. Kucherlapati, Shadi Esfahani, Peiman Habibollahi, Junning Wang, Eric R. Still, Roderick T. Bronson, Umar Mahmood, Raju S. Kucherlapati

**Affiliations:** 1 Department of Genetics, Harvard Medical School, Boston, Massachusetts, United States of America; 2 Department of Medicine, Division of Genetics, Brigham and Women’s Hospital Boston, Massachusetts, United States of America; 3 Department of Radiology, Division of Nuclear Medicine and Molecular Imaging, Massachusetts General Hospital, Boston, Massachusetts, United States of America; 4 Rodent Histopathology Core, Harvard Medical School, Boston, Massachusetts, United States of America; 5 Department of Pathology, Tufts University Schools of Medicine and Veterinary Medicine, Boston, Massachusetts, United States of America; National Institutes of Health, United States of America

## Abstract

The PI3K/AKT/mTOR pathway has frequently been found activated in human tumors. We show that in addition to *Wnt* signaling dysfunction, the PI3K/AKT/mTOR pathway is often upregulated in mouse *Msh2^−/−^* initiated intestinal tumors. NVP-BEZ235 is a dual PI3K/mTOR inhibitor toxic to many cancer cell lines and currently involved in clinical trials. We have treated two mouse models involving *Msh2* that develop small intestinal and/or colonic tumors with NVP-BEZ235, and a subset of animals with NVP-BEZ235 and MEK inhibitor ADZ4266. The disease phenotype has been followed with pathology, ^18^F FDG PET imaging, and endoscopy. Intestinal adenocarcinomas are significantly decreased in multiplicity by both drug regimens. The majority of tumors treated with combined therapy regress significantly, while a small number of highly progressed tumors persist. We have examined PTEN, AKT, MEK 1&2, MAPK, S6K, mTOR, PDPK1, and Cyclin D1 and find variable alterations that include downregulation of PTEN, upregulation of AKT and changes in its phosphorylated forms, upregulation of pMEK 1&2, p42p44MAPK, pS6K, and Cyclin D1. Apoptosis has been found intact in some tumors and not in others. Our data indicate that NVP-BEZ235 alone and in combination with ADZ4266 are effective in treating a proportion of colorectal cancers, but that highly progressed resistant tumors grow in the presence of the drugs. Pathways upregulated in some resistant tumors also include PDPK1, suggesting that metabolic inhibitors may also be useful in treating these tumors.

## Introduction

Colorectal cancer (CRC) is the third most frequently diagnosed cancer and cause of cancer mortality in the United States [1]. Therapeutic guidelines are based on TNM staging [2]. Stage I & II (low risk) invasive tumors are removed surgically, and patients with Stage II (high risk) & Stage III tumors are treated with adjuvant therapy that includes FOLFOX (5′-fluorouracil (5-FU)-leucovorin-oxaliplatin), FLOX (bolus 5-FU-leucovorin-oxaliplatin), and CapeOx (capecitabine-oxaliplatin). The use of monoclonal antibodies to VEGF-A (bevacizumab), EGFR (cetuximab & panitumumab), and topoisomerase inhibitors (irinotecan) are recommended for metastatic disease with appropriate genotype.

Because of the nonspecific nature of adjuvant therapies, their side effects, and the actual percentage of patients who derive benefit from them, it has long been hoped that the identification of the progressive changes to the genome would lead to the identification of points of intervention and the development of specific inhibitors that would be less damaging and cause fewer adverse events. Many of the major elements of the PI3K/AKT/mTOR pathway have been found mutated or amplified in a wide variety of tumor types [3] and for this reason PI3K inhibitors have been sought after. NVP-BEZ235 [4], is a compound with dual PI3K/mTOR inhibitor activity, that acts as an ATP competitor and has been shown to significantly reduce tumor growth in a number of human xenograft models [5]–[10] and PI3K driven lung models [11]–[15]. The drug is currently involved in clinical trials for solid tumors, breast cancer, endometrial cancer, and advanced renal cell carcinoma [16].

In this study we use two conditional mouse models bearing *Msh2* mutation that develop spontaneous intestinal or colonic tumors, to observe the effects of NVP-BEZ235. After identifying MEK upregulation with subsequent MAPK increase in a proportion of resistant intestinal adenocarcinomas, we have given MEK inhibitor ADZ6244 [17], in combination therapy with NVP-BEZ235. We follow effect of the drug(s) with both pathology, and ^18^F FDG PET as evidenced by focal increase of the ^18^F FDG that resolves with treatment. Molecular alterations in tumors of several different components of the PI3K/AKT/mTOR and MAPK/ERK signaling pathways as well as a metabolic indicator (PDPK1, synonymous with PDK1) have been examined, as has apoptosis. These data indicate that NVP-BEZ235 has efficacy in a proportion of tumors. However because of the possibility of increased progression in a subset of resistant tumors, and the diversity observed in signaling mechanisms, the drug should be used in combination therapy with other specific pathway inhibitors or conventional radiotherapy rather than alone when treating mismatch repair (MMR) deficient colorectal cancer.

## Materials and Methods

### Ethics Statement

All procedures were in strict accordance with the recommendations in the Guide for the Care and Use of Laboratory Animals of the National Institute of Health. All work reported here was under protocol # 03390, approved by Harvard Medical School’s Institutional Animal Care and Use Committee (IACUC). All surgery was performed under anesthesia, and all efforts were made to minimize suffering.

### *Msh2 ^LoxP/LoxP^ Cre* Recombinase Transgenic Mouse Lines

In the first model the conditional *Msh2* allele has been placed under the control of the *Villin-Cre* transgene (B6.SJL-Tg (Vil-cre) (97 Gum/J) [18], [19]. The majority of these animals develop adenocarcinomas of the small intestine.

### The Generation of Large Intestinal Tumors from *Apc^LoxP/LoxP^ Msh2 ^LoxP/LoxP^* Mouse Lines

In the second model large intestinal tumors were generated by infusing *Adenoviral-Cre* (Ad5CMVcre; Gene Transfer Vector Core University of Iowa) into the colons of mice bearing conditional alleles for both *Apc* and *Msh2* by laparotomy [20]. Animals were prepped the day before surgery by denuding abdominal skin with depilatory and replacing bedding with wire racks and fasting overnight. The next day, after anesthetizing with 2% isoflurane, a midline incision was made through the outer skin and the parietal peritoneum. Both layers were retracted with a wire speculum. The colon was clamped approximately three centimeters from the anus and PBS used to wash feces from the interior. A custom built catheter was used to deliver 100µL 0.05% trypsin to a specific area of the colon defined by placement of a second clamp. After ten minutes the second clamp was removed, the colon washed extensively with PBS, and the interior abraded mechanically using a miniature brush. A second catheter was placed and localized with another clamp for *Adenoviral-Cre* delivery. Virus was incubated *in vivo* for thirty minutes. Clamps were removed and the abdominal wall was sutured closed in two layers, peritoneum followed by the outer skin. Analgesics were administered after surgery (Buprenorphine, 0.05–0.1 mg/kg/SQ Q 8–12 h).

### *In Vitro* Drug Treatment of a Human CRC Cell Line

A CellTiter 96® Aq_ueous_ Assay (Promega) was used as described previously [21] to determine cell line sensitivity to NVP-BEZ235. Briefly, confluent plates of three human cell lines (HCT115, SW480, & LoVo) obtained from the American Type Culture Collection (ATCC) were treated with and suspended in RPMI-1640 10% fetal calf serum/1% Penicillin (HCT115), Leibovitz’s L-15 Medium (SW480), or F-12K Medium (LoVo). Cell viability was determined by trypan blue exclusion test. Cells were plated in quadruplicate in various concentrations for twenty-four hours. CellTiter 96® Aq_ueous_ Assay (Promega) one step reagent was added and after one hour the absorbance at 490 nm was read on an Elisa Plate Reader to determine the linear range of cell growth for each cell line. Cells were dispensed at this concentration subsequently to determine the effect of various doses of NVP-BEZ235, also by CellTiter 96® Aq_ueous_ Assay (Promega) one step reagent.

### *In Vivo* Drug Treatment

NVP-BEZ235-NX (hereafter called NVP-BEZ235) was obtained from Novartis Pharmaceuticals. Dry powder was dissolved in N-Methyl-2-pyrrolidone (NMP) with brief heating, followed by addition of PEG300 (1∶9 NMP/PEG). Drug was placed into solution immediately prior to delivery. Mice were weighed daily and given 25 mg/kg NVP-BEZ235/NMP/PEG by gavage for thirty days. ADZ4266 MEK 1&2 inhibitor was obtained from Selleck Chemicals. Dry powder was dissolved in NMP and PEG along with NVP-BEZ235. Mice were given 25 mg/kg ADZ4266 with NVP-BEZ235 five times per week by gavage for thirty days.

### Histopathologic Analysis

Tumors of the *Villin-Cre Msh2^−/−^* GI tract were examined systematically. After sacrifice the entire GI tract was removed. In four cases (two each, drug/control) intestinal “Swiss roles” were sectioned entirely, stained with Hematoxylin & Eosin (H&E), and tumors counted. All other intestines were opened longitudinally. Gross specimens were examined under a Zeiss dissecting microscope and the number and location of the tumors recorded. Tumors were fixed in 4% paraformaldehyde prior to being observed by pathology. Upon examination tumors were placed into four categories. A “grade 1” lesion was defined as an adenoma that floated above normal crypts, a “grade 2” lesion was an adenoma that grew down to the submucosa without invasion, a “grade 3” lesion was an adenocarcinoma that invaded into the mucosa and superficial muscularis, a “grade 4” adenocarcinoma invaded deeply into the muscularis. All tumors were examined by two individuals one of whom is an experienced veterinary pathologist. The Fisher exact probability test was used for the analysis of tumor incidence and tumor progression, and Mann-Whitney test used for tumor multiplicity.

### Positron Emission Tomography (PET) Imaging and Optical Colonoscopy

PET imaging was done on a subset of animals before and after drug delivery, for NVP-BEZ235 treated mouse models. The scan and subsequent image analysis were performed using an eXplore Vista microPET scanner (GE healthcare/Suinsa Medical Systems). All animals were fasted twelve to sixteen hours prior to imaging. Anesthesia was induced using 5% isoflurane and oxygen for 30 seconds, followed by 1.5% isoflurane and oxygen used for maintenance during both injection and imaging. [^18^F]-Fluoro-2-deoxy-D-glucose (^18^F FDG) was injected intravenously through the tail vein with approximately 500 µCi of activity. Mice were not anesthetized during the tracer accumulation period. Fifteen minute static whole body scans were acquired one hour after injection. The images were reconstructed using a two-dimensional ordered-subset expectation maximization algorithm (2D-OSEM); and correction was applied for attenuation and scatter. For mice treated with NVP-BEZ235 and ADZ4266 combined therapy, CT-PET images were obtained. PET imaging was acquired as described above. CT scanning was acquired in standard resolution. Scan parameters were as follows: tube voltage 40 kVp, tube current 140 µA, number of projections 360, number of shots 8, axial field-of-view 80 mm. Scans were recorded without respiratory gating. Total scan duration was about 14 minutes. Image data was reconstructed using FeldKemp algorithm.

For optical colonoscopy mice were anesthetized using 2% isoflurane. The colon was washed with PBS to cleanse the bowel. A custom made colonoscopy system was used as previously described [22]. Air was carefully insufflated into the colon to allow full visualization, but to avoid perforation. Endoscopic images and videos were saved for later offline analysis by Image J software [23].

### Immunohistochemistry and Western Blotting

Five micrometer sections were cut from tissue embedded in paraffin blocks for immunohistochemistry (IHC). Paraffin was removed using xylene and sections rehydrated. Antigen retrieval was done using 1X citrate buffer (pH6.0) (Zymed), and a Medical Decloaking Chamber (Biocare Medical). Sections were “blocked” for peroxidase (Dako), serum (Normal Donkey/Rabbit serum), and avidin and biotin (Vector Laboratories). Primary antibody was applied overnight at 4°C, biotinylated secondary antibody for thirty minutes at room temperature. A Vectastain ABC Kit (Vector Laboratories), followed by Liquid+Substrate Chromogen System (Dako) was used for antibody detection. Mayer’s Hematoxylin Solution and Scott’s Bluing Reagent were used for counterstaining. The following antibodies were obtained from Cell Signaling Technologies (Beverly, MA): AKTpan (cat. no. 4691, 1∶250 dilution), AKT-T308 (cat. no. 9275, 1∶800), AKT-S473 (cat. no. 4060, 1∶50), pMEK1&2 (cat. no. 2338S, 1∶50), p42p44MAPK (cat. no. 4377, 1∶100 and cat. no. 4376, 1∶400), pS6K (cat. no. 2215S, 1∶1000), Msh2 (cat. no. 2017S, 1∶200), mTOR (cat. no. 2983S, 1∶50), p-mTOR (cat. no. 2971, 1∶100). The following antibodies were obtained from Abcam Biotechnologies (Cambridge, MA): PTEN (cat. no. AB32199, 1∶250), PDPK1 (cat. no. AB109460, 1∶250), pPDPK1 (cat. no. AB31406, 1∶250). Antibody to Cyclin D1 was from Thermo-Scientific (cat. no. RM-9104-S1, 1∶50 dilution). Antibody to Apc was obtained from Gene Tex Inc. (cat. no. GTX15270, dilution 1∶250). Antibody to β-catenin was obtained from Santa Cruz Biotechnology, Inc. (cat. no. SC-1469-R, dilution 1∶250). Tunel and Caspase 3 analysis was done by the Special Immunohistochemistry Core Facility.

For Western blot analysis, protein concentration was determined using Bio-Rad Protein Assay (Bio-Rad). Lysate containing 10 and 25 milligrams of protein were separated on a 10% SDS/PAGE gel, and transferred to nitrocellulose membrane. After blocking with 1% BSA, the membrane was probed with primary antibody (two hours), and then by secondary antibody (one hour). Amersham™ ECL™ Western blot reagents (GE Healthcare) were used for detection. Some autoradiographs were screened by densitometry using Image J software, with one way ANOVA used to determine significance. Most autoradiographs were judged visually with no densitometry.

## Results

### *Msh2* Deficient Human Colorectal Cancer Cell Line is Sensitive to NVP-BEZ235

**Figure 1:**
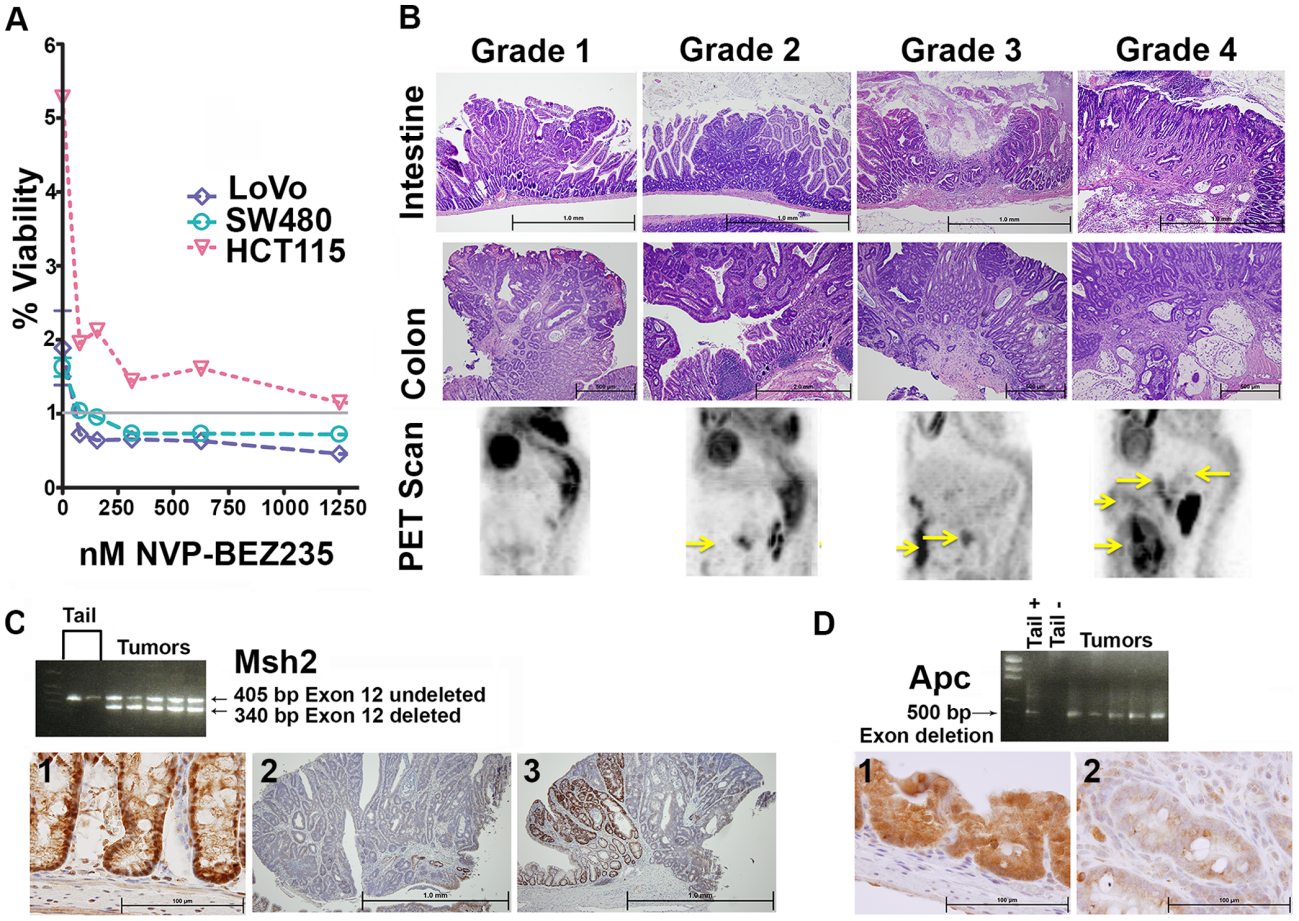
LoVo Sensitivity to NVP-BEZ235, *Msh2 & Apc* Gene Rearrangement, Tumor & PET Scanning Grading Criteria. (A) The effect of increasing amounts of NVP-BEZ235 on three human CRC cell lines is represented graphically. Percent viability = OD test sample/OD control sample×100, the grey line represents 100%. (B) Pictorial representation of Grades 1-4 for intestinal and colonic tumors and the graded scoring system used for PET scanning analysis of *Villin-Cre Msh2^−/−^* mice treated with NVP-BEZ235 alone. Grade 1 PET scan images have no abnormal radiotracer (^18^F FDG) uptake, Grade 2 have a single abnormal uptake focus, Grade 3 have multiple uptake foci or increased uptake in a loop of small intestine, and Grade 4 have both multiple uptake foci and increased uptake in a loop of small intestine. (C) *Msh2* gene rearrangement is identified by PCR analysis in five colonic adenomas from *Adenoviral-Cre Apc^−/−^ Msh2^−/−^* mice. 1 An IHC image using anti Msh2 antibody on colonic epithelium from a C57Bl6/J mouse. 2 A colonic adenoma from an *Adenoviral-Cre Apc^−/−^ Msh2^−/−^* mouse with complete Msh2 removal. 3 A colonic adenoma with partial *Msh2* removal. (D) *Apc* gene rearrangement is identified by PCR analysis in five colonic adenomas. 1 Anti Apc antibody on wildtype (C57Bl6/J) colonic epithelium. 2 Anti Apc IHC on a colonic adenoma.

Several human colorectal cancer cell lines have been found to be sensitive to NVP-BEZ235 however *MSH2* has been intact in those lines. To assess if *MSH2* mutant human colon cancer cells respond to this drug we examined LoVo (ATCC) and found this line to be sensitive to the drug as well, as determined by CellTiter96®AQ_ueous_Assay (Promega) (Fig. 1A).

### Altered Tumor Biology by NVP-BEZ235 Alone and in Combination Therapy with ADZ4266

#### Small Intestinal tumors

The effect of NVP-BEZ235 on tumor biology in *Villin-Cre Msh2^LoxP/LoxP^* mice was studied. Mice were treated with NVP-BEZ235 alone or with vehicle and compared to normal C57Bl6/J mice. The effect of NVP-BEZ235 in combined therapy with ADZ4266 was studied in an additional group. *Villin-Cre Msh2 ^LoxP/LoxP^* mice chosen for this study were seven months of age (seven months being the median survival rate for the colony) and clearly fecal occult blood (FOB) positive ensuring that intestinal tumors were present. Tumors were assigned a grade based on the degree of invasiveness. Grades 1 & 2 did not invade into the muscularis and were adenomas, while grades 3 & 4 had muscularis invasion and were therefore considered true adenocarcinomas (Fig. 1B). The results for tumor incidence and progression are presented in Tables 1 and 2. A decrease in the total neoplastic events was found in mice treated with both NVP-BEZ235 alone, and in combination with ADZ4266, the decrease was statistically significant in the combined therapy group. In both cohorts the drug resistant tumors are well advanced.

**Table 1:** Occurrence of carcinomas in drug treated small intestines.

Treatment	Sex	n	Total	Grade 1	Grade 2	Grade 3	Grade 4
Genotype (Age/Mo)	(M:F)		Neoplastic	n (%)	n (%)	n (%)	n (%)
			Events				
**Control/Vehicle treated**							
GVCM2CKO ^−/−^ (7–8)	4∶8	12	37^1, 3^	14 (37.9)	10 (27.0)	8 (21.6)^2^	5 (13.5)^4^
C57Bl6/J (4)		12	0	0	0	0	0
**NVP-BEZ235 treated**							
GVCM2CKO^−/−^ (7–8)	9∶3	12	27^1^	10 (37.0)	11 (40.8)	0 (0)^2^	6 (22.2)
C57Bl6/J (4)		12	0	0	0	0	0
**NVP-BEZ235 & ADZ6244**							
GVCM2CKO^−/−^ (7–8)	4∶8	12	19^3^	4(21.1)	2(10.5)	3(15.8)	10(52.6)^4^

1 Multiplicity by Mann-Whitney test, Control≠Drug P = 0.1005; Control >Drug P = 0.0503.

2 Mann-Whitney Control≠Drug P = 0.0145; Control >Drug P = 0.0072.

3 Mann-Whitney test, Control≠Drug P = 0.0121; Control >Drug P = 0.0006.

4 Mann-Whitney test, Control≠Drug P = 0.1600; Control >Drug P = 0.0800.

**Table 2:** Tumor progression in drug treated small intestines.

Adenocarcinomas	Control	NVP-BEZ235	NVP-BEZ235
			ADZ6244
**Total found (Grades 3 & 4)**	13^1^	6^1^	13
**No. (%) with histological verification**	13 (100)	6 (100)	13(100)
**Grade 3: n (% of total)**	8 (61.5)^2,3^	0 (0)^2^	3(23.1)^3^
**Grade 4: n (% of total)**	5 (38.5)^2,3^	6 (100)^2^	10(76.9)^3^

1 Multiplicity by Mann-Whitney test. Control≠Drug : P = 0.0387.

2 Fisher exact test Grades 3 vs 4, Control≠Drug : P = 0.0341.

3 Fisher exact test Grades 3 vs 4, Control≠Drug : P = 0.0302.

#### Colonic tumors

*Adenoviral-Cre* infusion into *Msh2^LoxP/LoxP^* mice did not lead to tumor generation. We were able to induce tumors in the large intestines of mice harboring conditional alleles for both *Apc* and *Msh2*. Seventy-one percent of the animals successfully infused had one to two large intestinal tumors, twenty-two percent had three to six tumors, and seven percent had too many tumors to count accurately. A size index for each tumor was calculated using a software measurement tool (Zeiss) that gave a range between three and twenty-three mm^2^. Colonic adenomas were generally large. Gene rearrangement of *Apc* and *Msh2* in several of these tumors was assessed by Polymerase Chain Reaction (PCR); and both genes were found to have been inactivated (Fig. 1C & D). Examination of tumor sections with IHC indicated that *Msh2* was not completely removed uniformly in all tumors (Table 3, Fig. 1C). *Apc* was removed uniformly in colonic adenomas (Table 3, Fig. 1D). Whole genome sequencing of colonic tumors (unpublished data) showed conclusively that both the *Apc* and *Msh2* were inactivated in adenocarcinomas. Sixty-five neoplastic events were examined by pathology in untreated mice. Tumors from these animals were placed into the same groupings (groups 1–4) as used for small intestinal tumors (Fig. 1B). Seventy-two percent of the neoplastic events were judged to be Grade 1, seventeen percent Grade 2, nine percent Grade 3, and two percent Grade 4. Adenomas made up 89% of the neoplastic events with 11% designated as adenocarcinomas. Seven mice were treated with NVP-BEZ235 and compared to two animals treated with vehicle. All of these mice were found to have drug resistant Grade 1 adenomas.

**Table 3:** *Wnt* and PI3K signaling deregulation in MMR deficient tumors by immunohistochemistry relative to normal small intestine and colon.

Antibody	Site (n) Treatment	Nuclear		Nuclear &		Membrane	
N = total				Cytoplasmic			
		(+)^1^	(–)^1^	(+)^1^	(–)^1^	(+)^1^	(–)^1^
		n (%)	n (%)	n (%)	n (%)	n (%)	n (%)
Msh2 N = 13^3^	Intestine (6) Untreated	0 (00)	6(100)				
	Intestine (7) NVP-BEZ235	0 (00)	7(100)				
Msh2 N = 13^3^	Colon (10) Untreated	6(60)^2^	4(40)				
	Colon (3) NVP-BEZ235	1(33)^2^	2(67)				
Apc N = 8^4^	Intestine (4) Untreated			1(25)	3(75)^2^		
	Intestine (4) NVP-BEZ235			0(00)	4(100)^2^		
Apc N = 5^4^	Colon (2) Untreated			0(00)	2(100)		
	Colon (3) NVP-BEZ235			1(33)	2(67)		
β-catenin N = 8^5^	Intestine (4) Untreated			4(100)	0(00)		
	Intestine (4) NVP-BEZ235			4(100)	0(00)		
β-catenin N = 5^5^	Colon (2) Untreated			0(00)	2(100)		
	Colon (3) NVP-BEZ235			0(00)	3(100)		
PTEN N = 9^6^	Intestine (6) Untreated			3(50)	3(50)		
	Intestine (3) NVP-BEZ235			1(33)	2(67)		
PTEN N = 6^6^	Colon (4) Untreated			0(00)	4(100)^2^		
	Colon (2) NVP-BEZ235			0(00)	2(100)^2^		
AKTpan N = 21^7^	Intestine (16) Untreated			15(94)^2^	1(6)		
	Intestine (5) NVP-BEZ235			5(100)^2^	0(00)		
AKTpan N = 5^7^	Colon (3) Untreated			3(100)	0(00)		
	Colon (2) NVP-BEZ235			2(100)	0(00)		

1 (+) Increased (or equivalent) staining relative to normal mucosa (–) decreased staining.

2 Nonuniform staining throughout the tumors.

3 Removal of *Msh2* is significantly better by *Villin-Cre* (small intestine) than by *Adenoviral-Cre* (colon),

Fisher exact P = 0.0026.

4 Apc is removed significantly in both tumor types relative to normal mucosa, Fisher exact P<0.0001.

5 β-catenin is significantly upregulated in adenocarcinomas of the small intestine but not in adenomas of the colon, Fisher exact P<0.0001.

6 The difference in PTEN decrease between the small and large intestinal tumors is not significant, Fisher exact P = 0.0923.

7 AKT is significantly upregulated in all tumors relative to normal mucosa, Fisher exact P<0.0001.

### PET Scans and Optical Colonoscopy Support Pathologic Findings in Both Mouse Models

#### Small intestinal tumors

**Figure 2:**
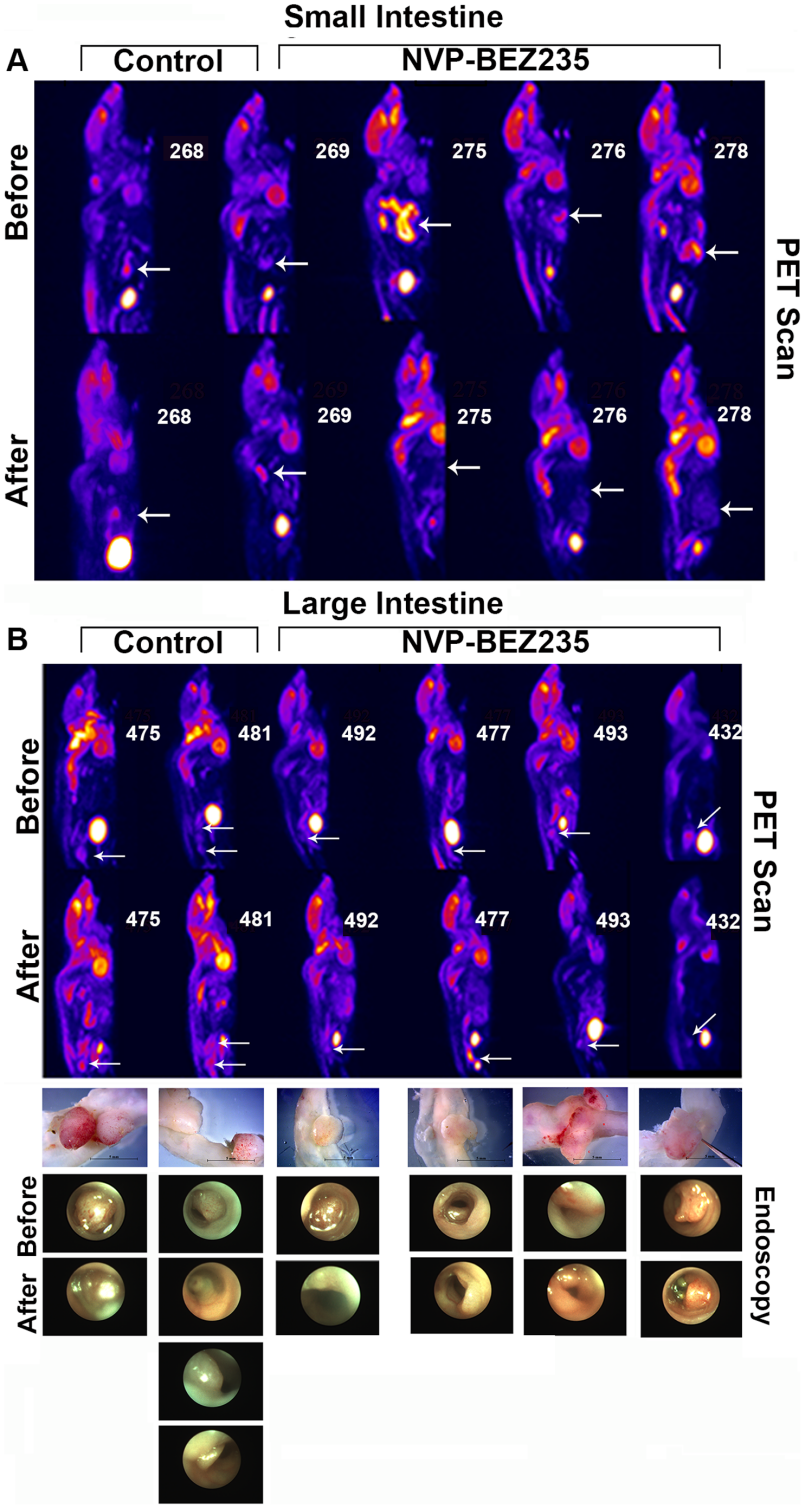
PET Scan Images Before and After NVP-BEZ235 Delivery in Two Mouse Models. (A) ^18^F FDG PET images of five *Villin-Cre Msh2^−/−^* mice. Arrows indicate abnormal foci of increased uptake with overall significant difference in “before” and “after” images. Signal with “increased uptake in loop” is seen in animal number 275 “before” image, no longer visible in the “after” drug treatment scan. (B) ^18^F FDG PET images of six *Adenoviral-Cre Apc^−/−^ Msh2^−/−^* mice with no overall significant difference in “before” and “after” images. Arrows indicate abnormal foci of increased uptake. Gross tumors obtained at autopsy are below the scans with images of tumors taken from colonoscopy videos made “before” and “after” treatment.

Because tumors occur predominantly in the small intestine in *Villin-Cre Msh2^LoxP/LoxP^* mice optical colonoscopy was not feasible, and they were screened with ^18^F FDG PET or CT-PET scans. A graded scoring system for PET scan signals from mice treated with NVP-BEZ235 alone (1 through 4) was developed and applied (Fig. 1B). Grade 1 had no abnormal radiotracer (^18^F FDG) uptake. Grade 2 had a single abnormal uptake focus. Grade 3 had multiple uptake foci or increased uptake in a loop of small intestine (Fig. 2A, Fig S1A), and Grade 4 had both multiple uptake foci and increased uptake in a loop of small intestine. We also examined two *Apc1638N* mice that have a phenotype of intestinal adenomas with ^18^F FDG PET and found only discreet signaling around the intestinal lesions (Fig. S1B), suggesting the more progressed tumors were responsible for the visualized “loop” signal. Comparison of a group of *Villin-Cre Msh2^LoxP/LoxP^* mice with only PET “after” drug/placebo images did not reveal a significant difference between drug and control groups (P = 0.119 by Mann-Whitney *U* test). However in a comparison of a second group of animals with both “before” and “after” drug/placebo delivery PET scan images, radiotracer uptake grades were pooled and the scores of “before” and “after” images judged significantly different (P = 0.031, independent samples; P = 0.034 drug treatment group) (Fig. 2A). In general, pathology and PET scans were in agreement for adenocarcinomas in the NVP-BEZ235 alone study. The intestines of four mice used for PET scans were placed into “Swiss rolls” and sectioned serially in their entirety. Very small early intestinal polyps were detected in this way and included in the pathology counts. These early polyps were not always picked up with PET scanning, potentially due to expected volume averaging of the imaging.

**Figure 3:**
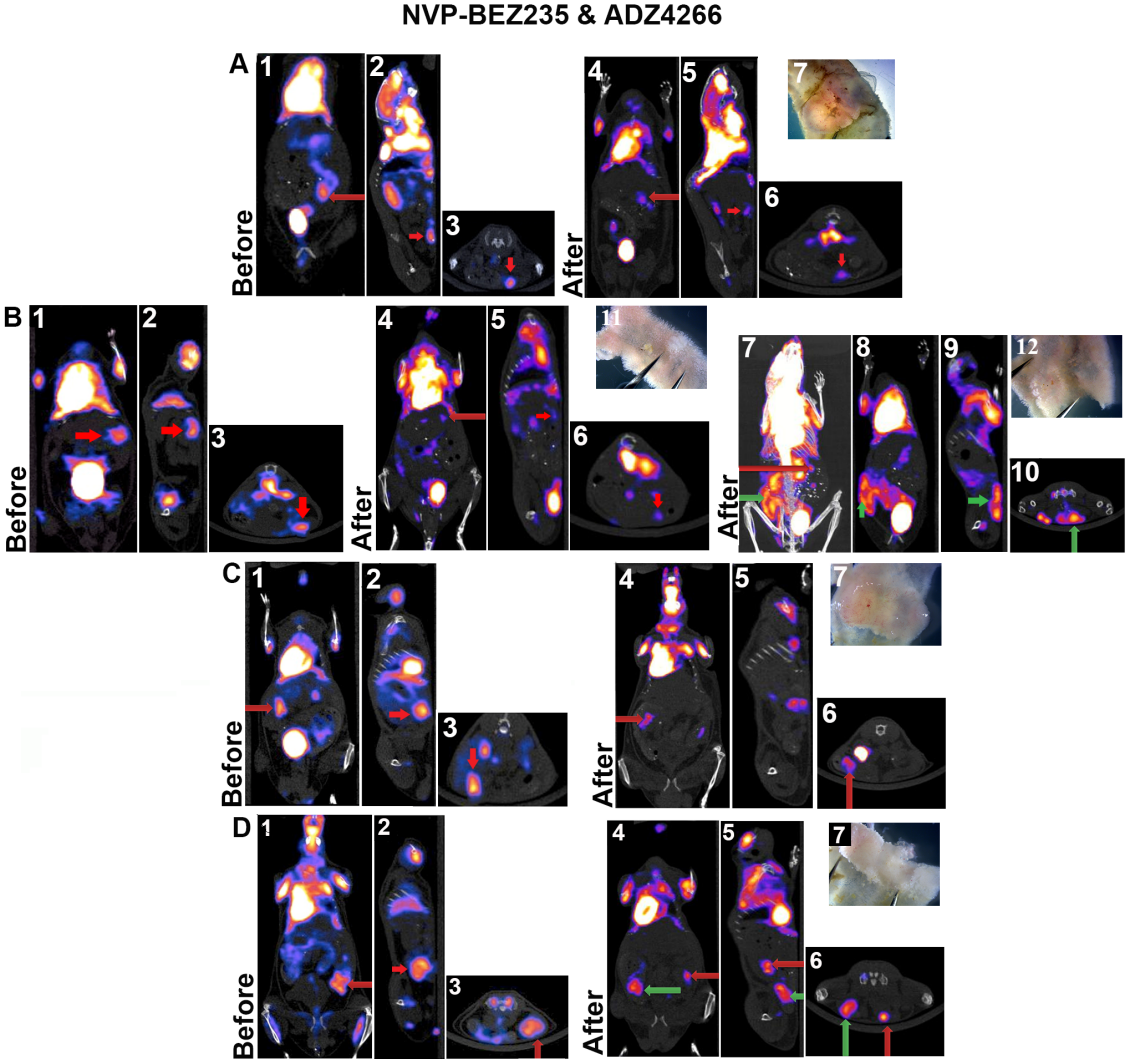
PET Scan Images Taken Before and After Combined NVP-BEZ235 and ADZ4266 Delivery in *Villin-Cre Msh2^−/−^* Mice. In four animals (A through D) treated with combined therapy, each had two tumors detected with ^18^F FDG CT-PET, the larger tumor is presented. (A) 1, 2, & 3, coronal, sagittal, and transverse images respectively are shown before drug treatment; (A) 4, 5, & 6, coronal, sagittal, and transverse images respectively are shown after drug treatment demonstrating regression, with the red arrow indicating tumor position. (A) 7, largest gross tumor found at necropsy (13.5 mg). (B) 1, 2, & 3 coronal, sagittal, and transverse images respectively are shown before drug treatment; (B) 4, 5, & 6, coronal, sagittal, and transverse images respectively are shown after drug treatment. The red arrow marks the tumor found in the “before” image (tumor regression), the green arrows in (B) 7, 8, 9, & 10/CT-PET, coronal, sagittal, transverse respectively, mark the development of a new resistant tumor. (B) 11, gross tumor found at necropsy (8 mg), (B) 12 a new drug resistant tumor (16 mg). (C) 1, 2, & 3, coronal, sagittal, and transverse images respectively are shown before drug treatment; (C) 4, 5, & 6, coronal, sagittal, and transverse images respectively are shown after drug treatment with tumor regression; (C) 7 shows the largest gross tumor at necropsy (205 mg). (D) 1, 2, & 3 coronal, sagittal, and transverse images are shown before drug treatment; (D) 4, 5, & 6 coronal, sagittal, and transverse images shown after drug treatment with tumor regression in one tumor and tumor development in the second (green arrows). (D) 7, largest gross tumor found at necropsy (230 mg).

For the NVP-BEZ235/ADZ4266 group of mice (Fig. 3 A–D) a standardized uptake value (SUV) for pre and post therapy was obtained that represented the ratio of signal obtained from the tumor and the cerebellum. The pre and post SUV maximum mean value for all tumors was 1.25±0.16 and 0.89±0.08 respectively (Paired t-test P = 0.012). Pre and post SUV mean value for all tumors was 0.7±0.05 and 0.58±0.04 (P = 0.025). Pre and post mean volume (cc) 0.32±0.1 and 0.075±0.03 (P = 0.045). Seventy-five percent of the tumors treated with combined therapy showed significant regression. The development of two resistant tumors was documented (Fig. 3B and D).

#### Colonic tumors

Adenomas of the large intestine created by *Adenoviral-Cre* infusion were examined both by optical colonoscopy (Fig. 2B, and Fig. S1C) and also by PET scan before and after drug/placebo delivery for the NVP-BEZ235 alone study (Fig. 2B). All animals, drug and control treated, had adenomas before and after drug/placebo treatment. Tumors in the control group exhibited progression. Evidence of partial regression was found in some NVP-BEZ235 treated animals, however this was not found significant. The pre and post SUV maximum mean value for these colonic tumors was 1.36±0.36 and 1.15±0.28 respectively (Paired t-test P = 0.61). The pre and post SUV mean values were 0.66±0.17 and 0.52±0.19 respectively (P = 0.36). Pre and post volumes (cc) were 0.12±0.99 and 0.06±0.05 (P = 0.35). Colonic tumor model images, while exhibiting ^18^F FDG uptake beyond the lesions, consistently had more discreet signals than the images from the intestinal model. While the apparent lack of drug efficacy correlated with decreased tumor progression seen in this model, the lack of significant decrease in values may have been due to sample size and a reflection of the relative difficulty in making the mice of that model.

### *Wnt* and PI3K/AKT/mTOR Signaling Pathways are Upregulated in MMR Deficient Tumors

**Figure 4:**
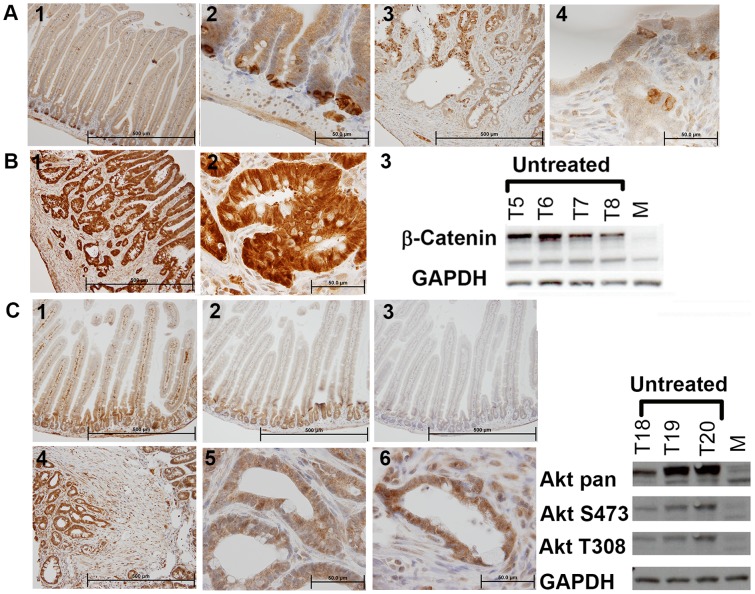
Apc and β-catenin Levels are Altered in *Villin-Cre Msh2^−/−^* Adenocarcinomas. (A) 1 & 2 anti Apc IHC on normal small intestinal sections. Panels 3 & 4 anti Apc IHC on adenocarcinomas from a *Villin-Cre Msh2^−/−^* mouse showing heterogeneous Apc expression with Apc loss in the invasive portion of the tumor. (B) 1 & 2, anti β-catenin IHC on an adenocarcinoma section from a *Villin-Cre Msh2^−/−^* mouse. 3 Western blot analysis of four *Villin-Cre Msh2^−/−^* adenocarcinomas showing increased β-catenin levels. (C) 1, 2, and 3, are IHC on normal mucosa using anti AKTpan, anti AKT-S473, and anti AKT-T308 respectively. 4, 5, and 6, show IHC on intestinal adenocarcinomas using anti AKTpan, anti AKT-S473, and anti AKT-T308 respectively. Western blot analysis of three adenocarcinomas showing increased total AKT and increased activated AKT (by phosphorylation) relative to normal mucosa.

Apc and β-catenin levels were examined in both mouse models. Apc was downregulated in the invasive regions of small intestinal tumors as shown by IHC, and β-catenin uniformly upregulated as shown by IHC and Western blot analysis (Table 3, Fig. 4A and B). Taken together, these findings are consistent with a large body of evidence that *Wnt* signaling pathway is deregulated in CRC.

To implicate NVP-BEZ235 as a rationale drug of choice for therapy in MMR deficient tumors, we examined specific alterations in AKT signaling pathway using IHC and/or Western blot analysis. AKT itself is a downstream effector of PI3K, and AKT phosphorylation in tumors has been associated with both poor and favorable outcomes in CRC [24]. In both our models total AKT levels (AKTpan) were found upregulated (Table 3, Fig. 4C). A proportion of tumors in both models had AKT phosphorylation at S473 and T308 (Table 4, Fig. 4C). The tumor suppressor PTEN that leads to upregulation of AKT was also found inactivated in a proportion of both tumor types (Table 3).

**Table 4:** Phosphorylation status of AKT and mTOR by immunohistochemistry.

Antibody	Site (n) Treatment	Nuclear		Nuclear &		Membrane	
N = total				Cytoplasmic			
		(+)^1^	(–)^1^	(+)^1^	(–)^1^	(+)^1^	(–)^1^
		n (%)	n (%)	n (%)	n (%)	n (%)	n (%)
AKT-T308 N = 21^3^	Intestine (13) Untreated			8(62)^2^	5(38)		
	Intestine (8) NVP-BEZ235			4(50)	4(50)		
AKT-T308 N = 13^3^	Colon (10) Untreated			9(90)	1(10)		
	Colon (3) NVP-BEZ235			3(100)	0(00)		
AKT-S473 N = 21^4^	Intestine (14) Untreated			6(46)^5^	8(54)^5^		
	Intestine (7) NVP-BEZ235			0(00)^5^	7(100)^5^		
AKT-S473 N = 13^4^	Colon (11) Untreated			11(100)^2^	0(00)		
	Colon (2) NVP-BEZ235			2(100)	0(00)		
mTOR N = 7^6^	Intestine (4) Untreated			4(100)	0(00)		
	Intestine (3) NVP-BEZ235			3(100)	0(00)		
mTOR N = 5^6^	Colon (3) Untreated			0(00)	3(100)		
	Colon (2) NVP-BEZ235			0(00)	2(100)		
p-mTOR N = 6^7^	Intestine (3) Untreated	3(100)^2^	0(00)				
	Intestine (3) NVP-BEZ235	3(100)^2^	0(00)				
p-mTOR N = 5^7^	Colon (3) Untreated			0(00)	3(100)		
	Colon (2) NVP-BEZ235			0(00)	2(100)		

1 (+) Increased (or equivalent) staining relative to normal mucosa (−) decreased staining.

2 Nonuniform staining throughout the tumors.

3 AKT-T308 is significantly downregulated in intestinal adenocarcinomas relative to colonic adenomas,

4 AKT-S473 is significantly downregulated in intestinal adenocarcinomas relative to colonic adenomas, Fisher exact P<0.0001.

5 AKT-S473 is downregulated in drug treated intestinal tumors relative to untreated intestinal tumors (staining judged more or less intense relative to normal crypts) Fisher exact P = 0.0553.

6 mTOR significantly upregulated in adenocarcinomas of the small intestine relative to adenomas of the colon, Fisher exact P = 0.0013.

7 p-mTOR localizes subcellular to the nucleus in adenocarcinomas and the cytoplasm of adenomas, Fisher exact P = 0.0022.

**Figure 5:**
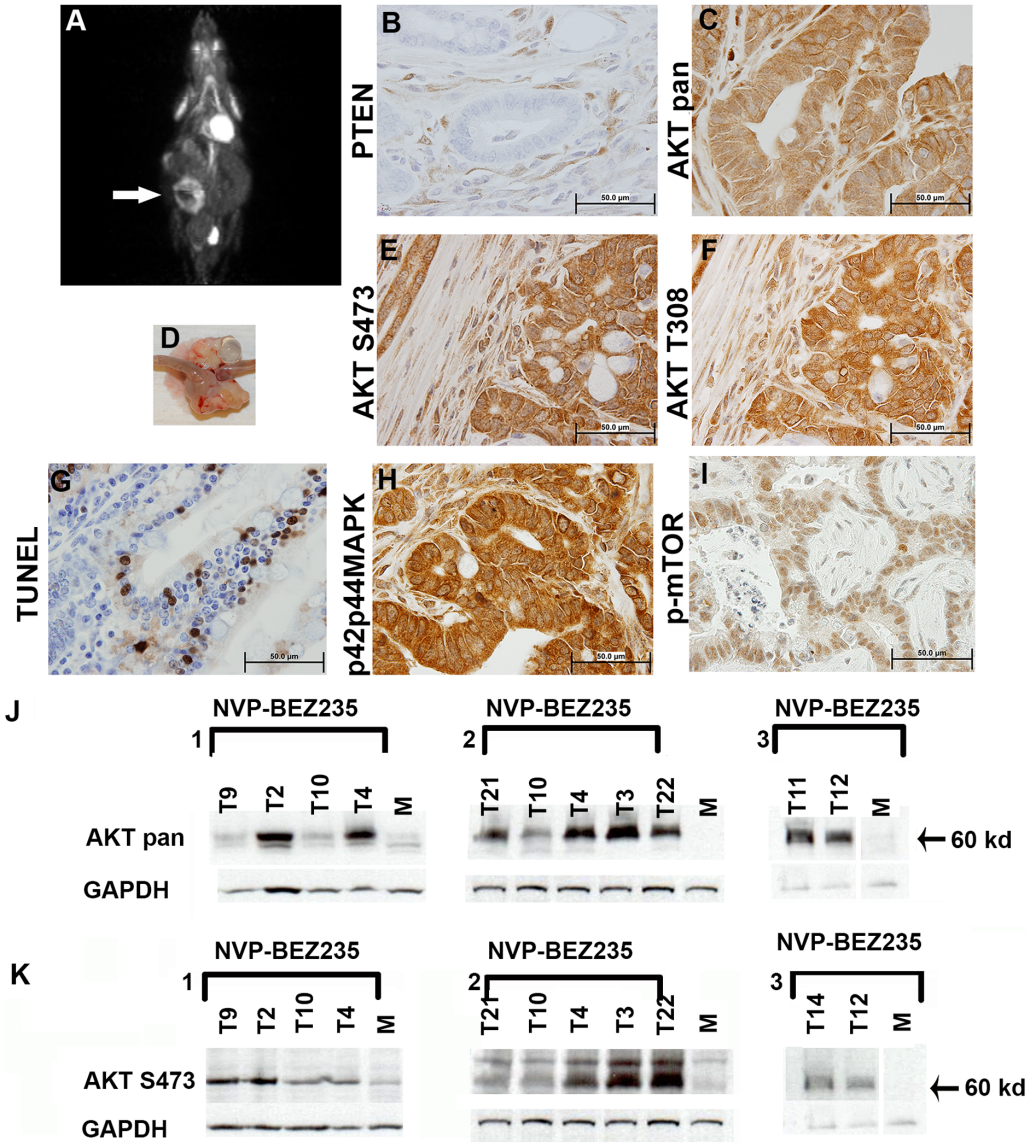
Intestinal Adenocarcinomas Resistant to NVP-BEZ235 Have Variable Levels of activated AKT, MAPK, and Apoptosis. (A) ^18^F FDG PET image of a *Villin-Cre Msh2^−/−^* mouse with NVP-BEZ235 resistant tumor with cyst (arrow). (B) IHC on section from tumor shown in panel “A” showing loss of PTEN in invasive cells. (C) IHC with anti AKT (AKTpan). (D) Gross tumor with cyst (E & F) IHC with anti AKT-S473 and anti AKT-T308, respectively. (G) Induction of apoptosis as seen with Tunel analysis (confirmed using anti Caspase 3). (H) IHC showing p42p44 MAPK upregulation (I) Activated p-mTOR is decreased in the cytoplasm and increased in the nucleus of the invasive portion of the adenocarcinoma. (J) Western blot analysis on NVP-BEZ235 resistant intestinal tumors (three separate blots presented). AKT protein levels differ between treated tumors (K) Western blot analysis showing AKT phosphorylation at S473 in drug resistant intestinal tumors.

**Figure 6:**
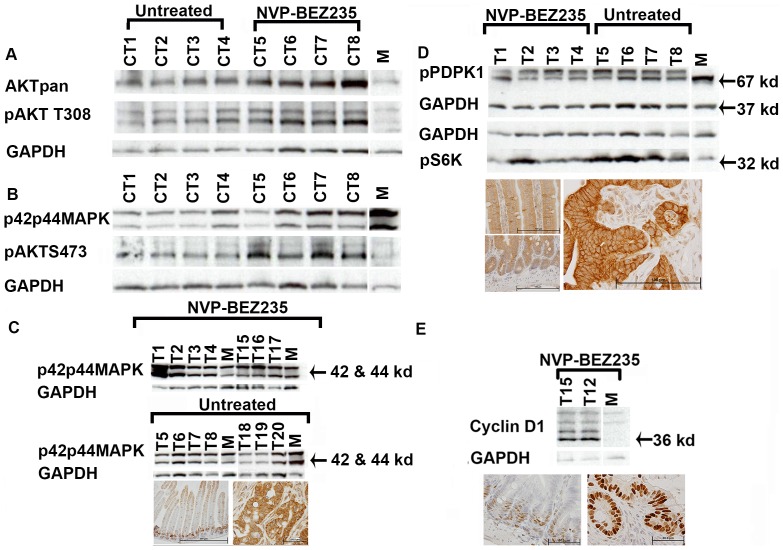
Molecular Analysis of Untreated and Drug Treated Tumors. (A and B) Western blot analysis of colonic adenomas (CT1–8) for total AKT levels (AKTpan), AKT activated by phosphorylation, and activated MAPK (“M” lanes, normal mucosa). (C) Small intestinal tumors examined for activated MAPK by Western blot analysis and IHC (left panel normal mucosa, right panel an invasive adenocarcinoma). (D) Western blot analysis of small intestinal adenocarcinomas and IHC using anti pPDPK1 showing increased membrane associated staining (left panel(s) normal mucosa, right panel an intestinal adenocarcinoma). (E) Western blot analysis and IHC for Cyclin D1 (left is normal mucosa, right is invasive adenocarcinoma).

Drug resistant tumors in both models were also examined by IHC (Tables 3–6, Fig. 5A–I) and Western blot analysis (Fig. 5 J & K, Fig. 6 A & B) in an initial attempt to explore drug efficacy, and trends in pathways between treated and untreated tumors. Overall IHC did not give definitive results when examining drug versus untreated groups. We did find by IHC that drug resistant tumors of the small intestine with increased total AKT had lowered AKT phosphorylation at S473 relative to untreated tumors, implying drug efficacy in some tumors but not in others (Table 4, Fig. 5K). We are assessing the basis for the difference. We find that p-mTOR known to be inhibited by NVP-BEZ235, localized to the nucleus of small intestinal adenocarcinomas and to the cytoplasm of adenomas independent of treatment (Table 4).

**Table 5:** MAPK/ERK signaling and metabolic deregulation by immunohistochemistry.

Antibody	Site (n) Treatment	Nuclear			Nuclear &		Membrane	
N = total					Cytoplasmic			
			(+)^1^	(–)^1^	(+)^1^	(–)^1^	(+)^1^	(–)^1^
			n (%)	n (%)	n (%)	n (%)	n (%)	n (%)
pMEK1&2^3 ^N = 11	Intestine (8) Untreated				8(100)	0(00)		
	Intestine (3) NVP-BEZ235				3(100)	0(00)		
pMEK1&2^3^ N = 5	Colon (3) Untreated				3(100)	0(00)		
	Colon (2) NVP-BEZ235				2(100)	0(00)		
p42p44MAPK^4,5^ N = 38	Intestine (21) Untreated				16(76)^2^	5(24)^2^		
	Intestine (17) NVP-BEZ235				16(94)^2^	1(6)^2^		
p42p44MAPK^4,5^ N = 13	Colon (10) Untreated				1(10)^2^	9(90)^2^		
	Colon (3) NVP-BEZ235				1(33)^2^	2(67)^2^		
pS6K N = 11^6^	Intestine (8) Untreated				8(100)^2^	0(00)		
	Intestine (3) NVP-BEZ235				3(100)^2^	0(00)		
pS6K N = 5^6^	Colon (3) Untreated				3(100)^2^	0(00)		
	Colon (2) NVP-BEZ235				2(100)^2^	0(00)		
PDPK1 N = 7^7^	Intestine (4) Untreated				4(100)	0(00)		
	Intestine (3) NVP-BEZ235				3(100)	0(00)		
PDPK1 N = 5^7^	Colon (3) Untreated				3(100)^2^	0(00)		
	Colon (2) NVP-BEZ235				2(100)^2^	0(00)		
pPDPK1 N = 7^8^	Intestine (4) Untreated						4(100)	0(00)
	Intestine (3) NVP-BEZ235						3(100)	0(00)
pPDPK1 N = 5^8^	Colon (3) Untreated						3(100)^2^	0(00)
	Colon (2) NVP-BEZ235						2(100)^2^	0(00)

1 (+) Increased (or equivalent) staining relative to normal mucosa (−) decreased staining.

2 Nonuniform staining throughout the tumors.

3 pMEK1&2 is significantly upregulated in all tumors relative to normal mucosa, Fisher exact P<0.0001.

4 p42p44MAPK is significantly upregulated in all tumors relative to normal mucosa, Fisher exact P<0.0001.

5 p42p44MAPK significantly upregulated in adenocarcinomas of the small intestine relative to adenomas of the colon, Fisher exact P<0.0001.

6 pS6K is upregulated (nonuniform staining) in all tumors relative to normal mucosa, Fisher exact P<0.0001.

7 PDPK1 is upregulated in all tumors relative to normal mucosa, Fisher exact P<0.0001.

8 pPDPK1 is significantly upregulated and membrane associated in all tumors relative to normal mucosa, Fisher exact P<0.0001.

**Table 6:** Cell cycle and apoptosis deregulation in MMR deficient tumors by immunohistochemistry.

Antibody	Site (n) Treatment	Nuclear		Nuclear &		Membrane	
N = total				Cytoplasmic			
		(+)^1^	(–)^1^	(+)^1^	(–)^1^	(+)^1^	(–)^1^
		n (%)	n (%)	n (%)	n (%)	n (%)	n (%)
Cyclin D1 N = 8^1^	Intestine (5) Untreated	5(100)	0(00)				
	Intestine (3) NVP-BEZ235	3(100)	0(00)				
Cyclin D1 N = 6^1^	Colon (3) Untreated	3(100)	0(00)				
	Colon (3) NVP-BEZ235	3(100)	0(00)				
Tunel N = 9^2^	Intestine (5) Untreated	0(00)^3^	5(100)^3^				
	Intestine (4) NVP-BEZ235	1(25)^3^	3(75)^3^				
Tunel N = 0	Colon (0) Untreated	n.d.^4^	n.d.				
	Colon (0) NVP-BEZ235	n.d.	n.d.				
Caspase 3 N = 8^2^	Intestine (5) Untreated	2(40)^5^	3(60)^5^				
	Intestine (3) NVP-BEZ235	2(67)^5^	1(33)^5^				
Caspase 3 N = 0	Colon (0) Untreated	n.d.	n.d.				
	Colon (0) NVP-BEZ235	n.d.	n.d.				

1 Cyclin D1 is up-regulated relative to normal mucosa in the nucleus of all adenocarcinomas and adenomas, Fisher exact P<0.0001.

2 Not significantly different, Fisher exact P = 0.30.

3 Not significantly different, Fisher exact P = 0.40.

4 “n.d.” not determined.

5 Not significantly different, Fisher exact P = 0.50.

### pMEK1&2, p42p44 MAPK, pS6K, PDPK1, Cyclin D1, Apoptosis Alterations in Tumors

pMEK1&2 were found significantly upregulated in all tumors relative to the normal mucosa by IHC (Table 5). This was true for p42p44MAPK as well, with confirmation by Western blot analysis in a proportion of intestinal tumors (Table 5, Fig. 6C). pS6K levels were also found upregulated (Table 5, Fig. 6D). The data are in correlation with the finding that MEK inhibitor ADZ4266 in combination with NV-BEZ235 was an effective drug regimen for a majority of intestinal tumors. Combination therapy has not yet been applied to the *Adenoviral-Cre* model.

The metabolic regulator PDPK1 can activate S6K by phosphorylation. All tumors examined with IHC had high PDPK1 signal localizing to the membrane with evidence of modification by phosphorylation in Western blot analysis (Table 5, Fig. 6D). All tumors showed upregulation of Cyclin D1 in the nucleus of invasive cells (Table 6). IHC results were confirmed with Western blot analysis (Fig. 6E).

Intestinal tumors, untreated or drug resistant, were examined for apoptosis using both TUNEL analysis and IHC with antibody to Caspase 3. We found apoptosis in some tumors (Table 6) but not others.

## Discussion

Human colorectal tumors can be classified as having high or low mutation rates [25]. About fifteen percent of CRC’s have high mutation frequencies with microsatellite instability, a feature known to be associated with defects in DNA mismatch repair resulting from mutation in MLH1, MSH2, PMS1, PMS2, or MSH6 [26]. We have treated MMR deficient murine adenocarcinomas of the intestine and adenomas of the colon with NVP-BEZ235, a PI3K/mTOR inhibitor, as a proportion of these tumors have alterations in the PI3K/AKT/mTOR signaling pathway. Upon identifying alterations to MAPK signaling pathway we have continued gene directed therapy using MEK inhibitor ADZ4266 in combination with NVP-BEZ235.

Both of the mouse models tested have *Msh2* deficiency, although the intestinal model has under lying molecular events that better mimic the sporadic human condition because *Msh2* deficiency acts as the driver for *Apc* loss. When the *Villin* promoter is in control of *Cre* recombinase the enzyme is constitutively expressed throughout development and in the adult gastrointestinal tract. The tumors are restricted to the small intestine. In the second model, *Adenoviral-Cre* infusion during laparotomy into the distal colons of three month old double homozygous *Apc^LoxP/LoxP^ Msh2 ^LoxP/LoxP^* mice enables reliable spontaneous adenoma formation in the large intestine that can be subsequently followed by endoscopy and other types of imaging without sacrificing the mice. The advantage to this model is that the tumors are induced by a somatic event and are located in the large intestine at the site of *Adenoviral-Cre* infusion. Both models provide different perspectives when evaluating the efficacy and safety of a cancer drug and in total give information that can help evaluate a drug for clinical use.

Using a rational approach to cancer therapy in our models we have shown that multiplicity and progression of tumors is affected by NVP-BEZ235 therapy alone and in combination with ADZ4266. Through imaging and pathology we have found evidence that some tumors were responsive to the drugs while others were resistant, suggesting the neoplastic pathways used by all of the tumors were not the same. The hypermutation phenotype associated with MMR deficiency may explain this heterogeneity. After NVP-BEZ235 treatment a proportion of resistant tumors are found to have upregulated MAPK signaling. This is in agreement with reports that suggest conditions of mTOR inhibition, as would occur with NVP-BEZ235 treatment, activate MAPK through RAS [27] and that cross talk exists between MAPK/ERK and PI3K/AKT/mTOR pathways. The addition of a MEK inhibitor in combined therapy with NVP-BEZ235 returns MAPK levels to predrug treatment levels in all tumors tested, as seen by Western blot analysis, but does not entirely eliminate the tumor phenotype. We have not yet investigated the mechanisms of resistance to combined therapy.

pS6K was heterogeneously upregulated in all tumors including NVP-BEZ235 treated tumors, suggesting that if a negative feedback inhibition loop with MAPK signaling pathway exists it was impaired in these tumors. Since PDPK1 can also activate S6K [28] we examined tumors from both groups for activated PDPK1 (pPDPK1), and found it highly upregulated by phosphorylation and recruited to the membrane. PDPK1 is thought to be a master regulator of both the AKT and MAPK pathways [29], [30], and may compensate for loss of PI3K when under selective pressure from the drug. PDPK1 is also involved in glucose metabolism and a body of still controversial evidence links the switch towards aerobic glycolysis in tumor cells with the upregulation of HIF-1α target genes (e.g.PDPK1, Glut1, Pfk1, Ldha) under the control of the histone deacetylase SIRT6 [31], [32]. Upregulation of phosphoglycerate dehydrogenase (PHGDH) as judged by IHC, an enzyme upstream of serine biosynthesis also occurs in our intestinal tumors (unpublished data) and to a lesser extent in the colonic adenomas. Serine is the precursor to glycine that is essential for purine biosynthesis thought to supply ATP synthesis in the absence of the Tricarboxylic Acid Cycle (TCA) cycle [33], [34], and thought to supply the increased need for Phosphatidylserine (PS) in membrane biogenesis of rapidly proliferating tumor cells. It is being rediscovered as a potential target for intervention, having been proposed as such for many years [35]. New inhibitors are becoming available for targeting metabolism, and because all adenocarcinomas seem to have the metabolic shift towards aerobic glycolysis it may also be a good point of intervention.

## Supporting Information

Figure S1 **Additional PET Scan and Colonoscopy Images. (**A) ^18^F FDG PET image of a *Villin-Cre Msh2^−/−^* mouse showing increased uptake in morphologically normal intestine proximal to an adenocarcinoma. (B) PET scan images of a mouse with *Apc1638N* genotype using ^18^F FDG tracer, an intestinal lesion (adenoma) is indicated by a yellow arrow in the anterior middle abdomen. There is physiologic ^18^F FDG uptake in the heart (brown arrow) and in brown fat (red arrow), and thoracic musculature (blue arrow). (C) Optical colonoscopy on mice with *Apc ^loxP/LoxP^ Msh2 ^loxP/loxP^* genotype, shows tumors before and after NVP-BEZ235 drug treatment.
(TIF)
