# Supplementary figures and images for: Genotype Directed Therapy in Murine Mismatch Repair Deficient Tumors
Source: PLoS One. 2013 Jul 23;8(7):e68817. doi: 10.1371/journal.pone.0068817 (PMC3720855; doi:10.1371/journal.pone.0068817)

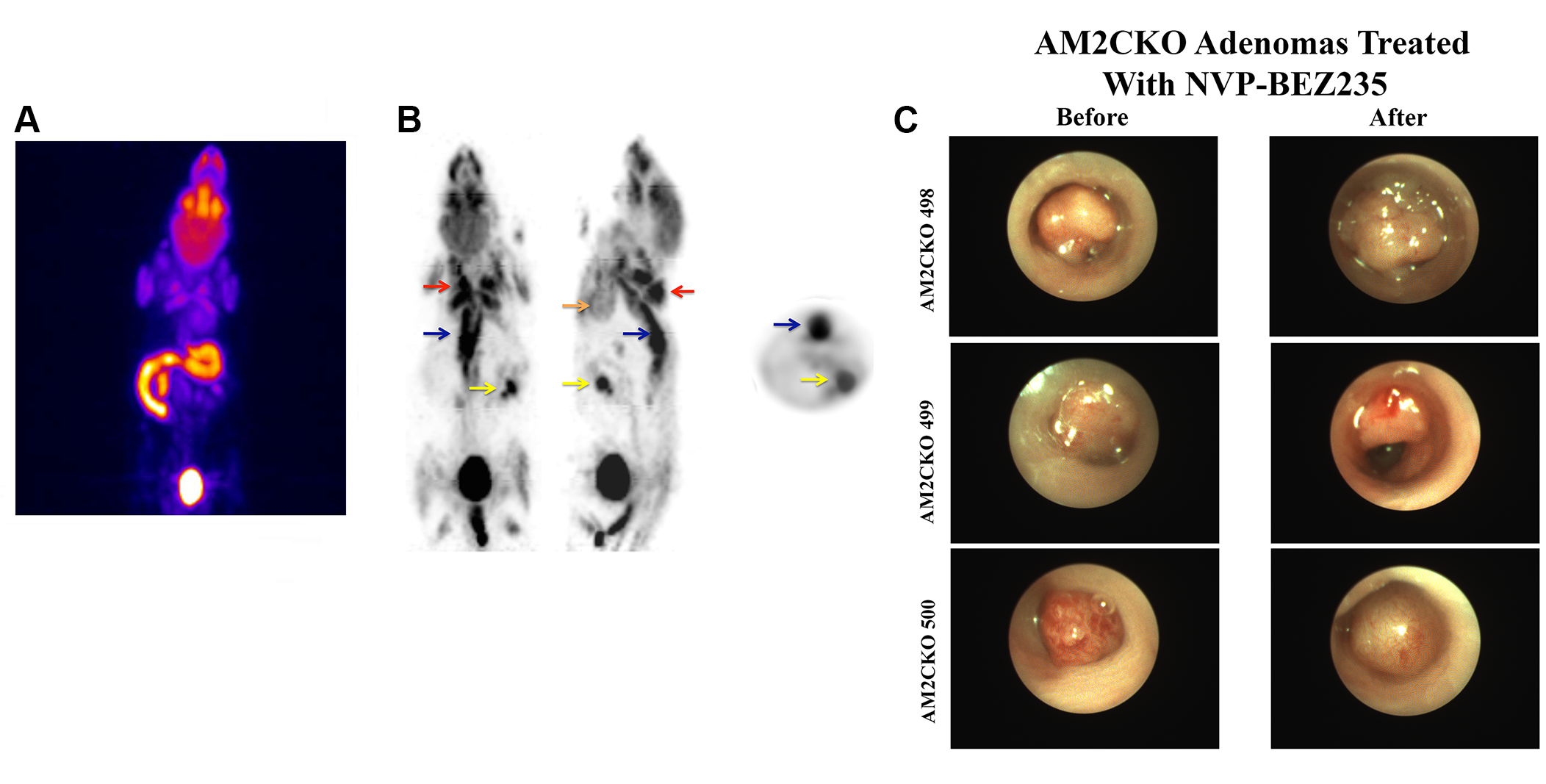

Supplement: Figure S1 — Additional PET Scan and Colonoscopy Images. (A) 18F FDG PET image of a Villin-Cre Msh2−/− mouse showing increased uptake in morphologically normal intestine proximal to an adenocarcinoma. (B) PET scan images of a mouse with Apc1638N genotype using 18F FDG tracer, an intestinal lesion (adenoma) is indicated by a yellow arrow in the anterior middle abdomen. There is physiologic 18F FDG uptake in the heart (brown arrow) and in brown fat (red arrow), and thoracic musculature (blue arrow). (C) Optical colonoscopy on mice with Apc loxP/LoxP Msh2 loxP/loxP genotype, shows tumors before and after NVP-BEZ235 drug treatment. (TIF) [file pone.0068817.s001.tif]
